# New insights in transcriptional control of somatic embryogenesis: the role of AGAMOUS-like 18

**DOI:** 10.1093/plphys/kiab593

**Published:** 2022-03-04

**Authors:** Elisa Dell’Aglio

**Affiliations:** Institute National des Sciences Appliquées de Lyon, 69100 Villeurbanne, France

In the appropriate conditions, differentiated cells within plant organs, such as roots and leaves, can dedifferentiate and produce new plant embryos. This process, called “somatic embryogenesis” is of high economic importance, as it allows fast propagation of desirable genotypes in crops such as coffee (*Coffea arabica*), cacao (*Theobroma cacao*), and poplar (*Populus trichocarpa*). Moreover, somatic embryogenesis can speed up the regeneration of mutants, especially for slow-growing plant species. However, various plants of economic value (including several varieties of soybean—*Glycine max*) are recalcitrant to somatic embryogenesis and a better understanding of this process could therefore help engineering tailored solutions for plant regeneration (Meurer et al., 2001).

Recent research has revealed several transcription factors (TFs) able to promote somatic embryogenesis to various extents. Many of them are redundant and involved in the same regulatory circuits. Among these TFs, the Arabidopsis (*Arabidopsis thaliana*) AGAMOUS-like 15 (AGL15) is a MADS-box-containing protein known to directly bind DNA (normally as a dimer) and orchestrate transcription of its targets, including a cluster of three core genes all necessary and sufficient for the process of somatic embryogenesis (Leafy Cotyledon 2—*LEC2*, FUSCA3—*FUS3*, and ABA insensitive 3—*ABI3*). As a consequence, AGL15 overexpression leads to an increase in somatic embryogenesis events (Zheng et al., 2009).

In this issue of *Plant Physiology*, Priyanka Paul and colleagues from the University of Kentucky, USA, investigate the crosstalk between AGL15 and its homolog AGL18, another MADS-box containing protein sharing 42% identity with AGL15. Although AGL18 has never been experimentally proven to directly bind DNA, its overexpression is accompanied with increased somatic embryogenesis in soybean (Zheng and Perry, 2014), and therefore it was expected that AGL18 also would play a direct or indirect role in promoting somatic embryogenesis via transcriptional regulation.

Firstly, the authors confirmed the putative role of AGL18 in promoting somatic embryogenesis in Arabidopsis by showing that *AGL18* overexpression doubles the somatic embryogenesis events in germinating seeds exposed to the synthetic auxin analog 2,4D. Vice versa, the double mutant *agl15 agl18* showed the opposite phenotype, with a greater than 5% reduction of somatic embryogenesis events. The absence of phenotype in the single *agl15* and *agl18* mutants strengthened the hypothesis of redundancy for these two TFs.

Indeed, coimmunoprecipitation of AGL15 and AGL18 demonstrated direct binding between the two proteins, suggesting that they can interact in vivo and conjunctly regulate gene expression. Point mutations mimicking phosphorylation (X -> D substitutions) led to a 10% increase in somatic embryogenesis events, as already previously observed for AGL15 (Patharkar et al., 2016). Vice versa, point mutations blocking phosphorylation (X -> A substitutions) led to a reduction in somatic embryogenesis events. These results further support a complementary role for AGL15 and AGL18 and suggest phosphorylation as a major mechanism of regulation for the AGL15/AGL18 pathway.

ChIP-Seq and RNAseq approaches on *AGL18-* or *AGL15*-overexpressing lines allowed a comprehensive mapping of AGL15 and AGL18 binding loci and target genes. Interestingly, the authors found 9729 and 3446 genomic loci associated to *AGL15* and *AGL18* overexpression, respectively. The 1254 shared loci were mostly associated with gene ontology categories related to post embryonic development and gene expression regulation, while *AGL18*-specific loci were mainly linked to seed development and plant ovule morphogenesis categories and *AGL15*-specific loci were mainly linked to hormonal responses.

Interestingly, RNAseq results revealed that several hundred genes were differentially regulated by *AGL18* overexpression, but less than 10% of these genes were also physically associated with the AGL18 protein, according to ChIP-Seq analyses. This discrepancy between direct (or indirect) promoter binding and transcriptional regulation might be due to the technique used (ChIPseq requires crosslinking with formaldehyde, which tends to increase the number of false positives) but also to the high redundancy of the regulatory circuits that govern somatic embryogenesis, which likely require a plethora of other regulatory components to drive (or repress) transcription besides AGL15/AGL18.

Validation by reverse transcription quantitative PCR (RT-qPCR) and ChIP-qPCR experiments confirmed both the transcriptional regulation in response to *AGL18* overexpression as well as physical association of AGL18 to the promoter region of a subgroup of candidate genes, including the gibberellin biosynthesis gene *GA3OX2* (Curaba et al., 2004). Notably, *GA3OX2* is downregulated in response to *AGL18* overexpression and is induced in *agl15* mutants (Zheng et al., 2009). By analyzing a *ga3ox2* mutant line, the authors observed an increase in somatic embryogenesis, thus confirming that gibberellins repress somatic embryogenesis and that their production is controlled by AGL15/AGL18.

**Figure 1:**
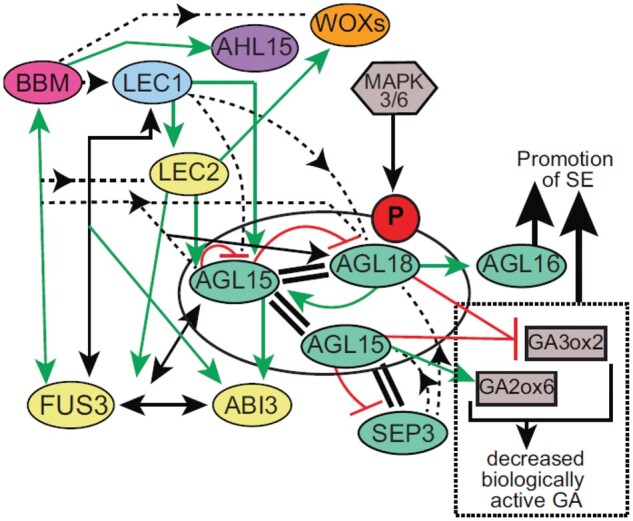
Working model showing interactions between the major actors driving somatic embryogenesis. Ovals indicate TFs, solid green arrows and red bars indicate direct upregulations and downregulations, respectively. Dotted lines indicate putative associations with regulatory gene regions and arrowheads indicate TFs binding each gene. Protein–protein interactions are shown as double bars. Oval around AGL15 and AGL18 indicates phosphorylation. (From Paul et al., 2021, Figure 9). P, phosphorylation; GA, gibberellin; SE, somatic embryogenesis.

Another direct target of AGL18 also confirmed at the transcriptomic level was *AGL15* itself. A deeper analysis of cross-transcriptional regulation between AGL15 and AGL18 using overexpressing lines and loss-of-function mutants revealed that AGL15 represses the transcription of *AGL18*, while AGL18 promotes *AGL15* transcription. Thus, AGL18 transcription could regulate itself by a negative feedback loop by promoting *AGL15* transcription that in turn represses both *AGL15* (Zhu and Perry, 2005) and *AGL18* transcription (Figure  1).

In conclusion, this work shed important light on AGL15/AGL18 roles in orchestrating somatic embryogenesis at the genomic scale. To further extend these results and boost applications of somatic embryogenesis for the regeneration of recalcitrant crops, it will be important to better characterize the transcriptional regulatory complexes of AGL15/AGL18 by CoIP followed by proteomics and by structural approaches for protein supercomplexes, such as cryoEM, on a variety of plant species.

*Conflict of interest statement*. None declared.
